# Intestinal Barrier Dysfunction and Gut Microbiota in Non-Alcoholic Fatty Liver Disease: Assessment, Mechanisms, and Therapeutic Considerations

**DOI:** 10.3390/biology13040243

**Published:** 2024-04-06

**Authors:** Changrui Long, Xiaoyan Zhou, Fan Xia, Benjie Zhou

**Affiliations:** 1 Department of Pharmacy, The Seventh Affiliated Hospital of Sun Yat-sen University, Sehenzhen 518107, China; longchr@gdmu.edu.cn; 2 School of Pharmacy, Guangdong Medical University, Dongguan 523808, China; 3 Department of Cardiovascular, The Seventh Affiliated Hospital of Sun Yat-sen University, Shenzhen 518107, China; zhouxiaoyan@sysush.com; 4 Shenzhen Key Laboratory of Chinese Medicine Active Substance Screening and Translational Research, Shenzhen 518107, China

**Keywords:** intestinal barrier dysfunction, gut dysbiosis, probiotics, NAFLD/NASH

## Abstract

**Simple Summary:**

Non-alcoholic fatty liver disease (NAFLD) is a type of metabolic stress liver injury closely related to insulin resistance (IR) and genetic susceptibility without alcohol consumption, which encompasses a spectrum of liver disorders ranging from simple hepatic lipid accumulation, known as steatosis, to the more severe form of steatohepatitis (NASH). In this narrative review, we summarize the roles of gut barrier dysfunction and gut microbiota in NAFLD, including their physiological functions, assessment methods, related mechanisms, and therapeutic approaches for the prevention and treatment of NAFLD.

**Abstract:**

Non-alcoholic fatty liver disease (NAFLD) is a type of metabolic stress liver injury closely related to insulin resistance (IR) and genetic susceptibility without alcohol consumption, which encompasses a spectrum of liver disorders ranging from simple hepatic lipid accumulation, known as steatosis, to the more severe form of steatohepatitis (NASH). NASH can progress to cirrhosis and hepatocellular carcinoma (HCC), posing significant health risks. As a multisystem disease, NAFLD is closely associated with systemic insulin resistance, central obesity, and metabolic disorders, which contribute to its pathogenesis and the development of extrahepatic complications, such as cardiovascular disease (CVD), type 2 diabetes mellitus, chronic kidney disease, and certain extrahepatic cancers. Recent evidence highlights the indispensable roles of intestinal barrier dysfunction and gut microbiota in the onset and progression of NAFLD/NASH. This review provides a comprehensive insight into the role of intestinal barrier dysfunction and gut microbiota in NAFLD, including intestinal barrier function and assessment, inflammatory factors, TLR4 signaling, and the gut–liver axis. Finally, we conclude with a discussion on the potential therapeutic strategies targeting gut permeability and gut microbiota in individuals with NAFLD/NASH, such as interventions with medications/probiotics, fecal transplantation (FMT), and modifications in lifestyle, including exercise and diet.

## 1. Introduction

The prevalence of non-alcoholic fatty liver disease (NAFLD) has become a significant public health issue worldwide [1,2]. NAFLD encompasses a range of liver disorders, beginning with the harmless accumulation of fats in the liver, termed steatosis, and potentially advancing to more severe stages such as non-alcoholic steatohepatitis (NASH), fibrosis, and, in extreme cases, cirrhosis [3,4]. The development of concurrent problems like systemic vascular endothelial dysfunction, atherosclerosis, and reproductive disorders is associated with an increased risk of NAFLD [5,6,7,8]. In recent years, much research has established a correlation between NAFLD and various non-liver systemic comorbidities, including heart disease, type 2 diabetes, kidney problems, and neurological disorders such as depression, anxiety, and apathy [9,10]. In 2023, three major multinational liver associations proposed that the term metabolic dysfunction-associated steatotic liver disease (MASLD) should replace NAFLD, and metabolic dysfunction-associated steatohepatitis (MASH) should replace NASH, underscoring the critical role of abnormal metabolism in the pathogenies of these conditions [11].

The progression of NAFLD is a complex phenomenon involving intricate molecular and environmental interactions. The “two-strike hypothesis” has previously been used to describe the development of NAFLD, where the first impact is the accumulation of fats in the liver due to factors such as a diet high in fat, overweight/obesity, and insulin resistance, followed by inflammatory cytokines, adipokines, mitochondrial malfunction, and oxidative stress [12]. However, this hypothesis appears insufficient to explain the metabolic and molecular changes occurring in NAFLD hepatocytes. The “multiple-hit” hypothesis has emerged as a more comprehensive explanation, suggesting that multiple factors act together to induce NAFLD in genetically susceptible populations [1,13,14]. These factors influence the inflammatory and stress environment within hepatocytes, leading to metabolic dysfunction. Recent research has unveiled the pivotal role of intestinal barrier dysfunction and gut microbiota in the initiation and progression of NAFLD/NASH [15]. The concept of the “intestinal barrier” involves a complex interplay of structural and functional mechanisms, including the gut microbiota barrier and mucus, gastrointestinal motility and secretion, epithelial barrier, immune system (both innate and adaptive), gut vascular system, and the liver barrier [16]. Dysfunction in any part of the intestinal barrier is a key factor in the pathogenesis of NAFLD. However, the specific contributions of the intestinal barrier and gut microbiota to NAFLD and the associated mechanisms are not yet fully understood. Therefore, we conducted a comprehensive search of various electronic databases, including g Google Scholar, PubMed, ScienceDirect and Medline, using multiple keywords (NAFLD/NASH, intestinal/gut barrier, microbiota, assessment, probiotics, TLR4, inflammation, etc.). In the review, we describe the intestinal barrier dysfunction and assessment, as well as gut microbiota in individuals with NAFLD, supporting the potential use of interventions targeting the restoration of intestinal barrier integrity and gut microbiota as a treatment strategy for managing the condition.

## 2. Function and Composition of the Intestinal Barrier

**Figure 1:**
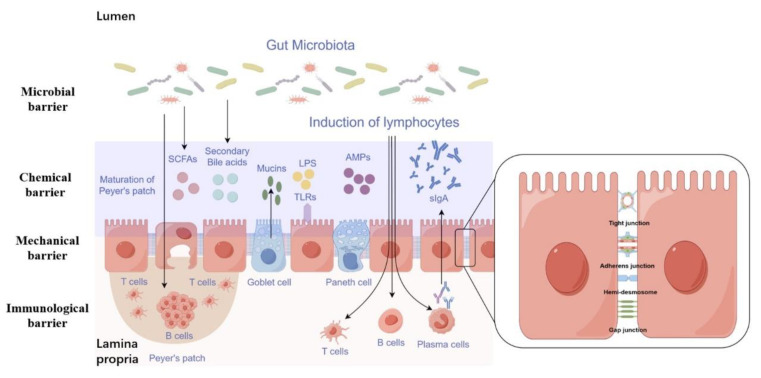
Schematic representation of the main components of the intestinal barrier. The intestinal barrier is a semi-permeable structure facilitating the absorption of essential nutrients and immune surveillance, while at the same time preventing the entry of pathogenic substances and bacteria. The mucus layer acts as a filter-like barrier covering the intestinal epithelium, containing antimicrobial peptides (AMPs), secretory IgA molecules (sIgA), lipopolysaccharides (LPSs), and secondary bile acids. Intestinal epithelial cells (IECs) form a continuous monolayer that is tightly interconnected through apical junctional complexes. Tight junctions (TJs) located at the apical surface regulate the passage of small molecules and ions. Adherens junctions (AJs) and desmosomes provide strict cell adhesion bonds and help maintain the integrity of the intestinal barrier. Gap junctions facilitate the exchange of both chemical and electrical signals between neighboring cells. Within the lamina propria reside immune cells from both the adaptive and innate immune system that play active roles in defending the intestinal barrier against various threats.

Maintaining epithelial and endothelial barriers in various body parts, including the gut, skin, blood vessels, respiratory tract, and the brain, is essential for human health. The intestine represents the largest and one of the most crucial internal barriers in the body, shielding the host from harmful substances and microorganisms within the gut lumen [17]. The gastrointestinal (GI) mucosa is a semi-permeable barrier with numerous functions, including nutrient absorption and immune detection [18]. The intestinal barrier consists of the gut microbiota, mucus layer, epithelial cells monolayer, and immune cells within the lamina propria, transitioning from the innermost luminal layer to the outer basolateral layer (Figure 1) [17]. The intestinal barrier is classified into four main categories based on its functional classification: (1) mechanical barrier, (2) chemical barrier, (3) microbial barrier, and (4) immune barrier [19].

### 2.1. Mechanical Barrier

The anatomical basis of the intestinal mechanical barrier derives from the connections between the intestinal epithelial cell (IEC) layer and adjacent cells [20,21]. These connections are critical for maintaining intestinal permeability in NAFLD. The apical junctional complex (AJC), which includes tight junctions (TJs) and adherens junctions (AJs), is primarily composed of neighboring cells [22]. IEC requires AJC assembly, and AJC, in turn, requires anchoring by IEC. The two functions cannot exist in isolation, indicating that the integrity of IEC and the stability of AJC play significant roles in regulating intestinal permeability in NAFLD. In addition, there are also various structures for the adhesion of epithelial cells to the lamina propria, including the hemidesmosomes and gap junctions [17].

Tight junctions (TJs), located at the apex of the AJC, are crucial for preserving intestinal permeability. TJs are composed of membrane proteins such as Claudin, Occludin, and Junctional Adhesion Molecules (JAMs), along with peripheral cytoplasmic proteins like Zonula Occludens-1 (ZO-1), ZO-2, and ZO-3 [23]. The level of tight junction proteins is inversely related to intestinal permeability [24], indicating a vital role in maintaining barrier function. The structural stability of TJs is dependent on their anchoring to cytoskeletal proteins. The ZO-1 protein interacts extensively with cytoskeletal proteins and is essential for assembling and stabilizing AJC by anchoring to cytoskeletal proteins. Consequently, alterations in TJ performance are closely associated with ZO-1 protein [25], which is commonly used as a marker for TJ permeability and significantly influences both the formation and functional modifications of AJC. The positions of AJs are located below TJs and play a vital role in maintaining the integrity of the IEC and facilitating intercellular coordination, thereby determining the adhesive capability of the IEC [26]. The components of AJs include E-cadherin, γ-catenin, α-catenin, and p120-catenin, with E-cadherin playing a pivotal role in the maintenance of AJ structures. Nano-mechanical measurements have shown that E-cadherin is capable of detecting gaps within fibrous actin (F-actin) cytoskeletal protein fibers, thereby facilitating the anchoring of cytoskeletal proteins and enhancing the stability of the AJC [27]. The equilibrium between E-cadherin polymerization and dissociation is essential for preserving cellular morphology and organization. Decreased expression of E-cadherin has been linked to heightened hypoxia-induced IEC apoptosis, while increased expression has been shown to have the opposite effect [28]. Disruption of the balance between E-cadherin polymerization and dissociation leads to structural changes in E-cadherin and the disintegration of AJs [29,30], resulting in decreased intercellular adhesion, which not only leads to cell detachment and apoptosis but also interferes with the barrier function of TJs.

### 2.2. Chemical Barrier 

The intestinal chemical barrier serves to protect the integrity of the intestinal lining by preventing the intrusion of microorganisms and enzymes [31]. This barrier consists of various components, including stomach acid secretions, mucus, mucins, bile, bile acids, glycoproteins, mucopolysaccharides, digestive enzymes, lysozymes, and antimicrobial peptides. Antimicrobial peptides are specialized molecules capable of eliminating microorganisms while withstanding the harmful effects of host bacteria and pathogen, thereby maintaining the integrity and function of the intestinal barrier [32]. Stomach acid is essential in separating bacteria from the digestive system and preventing microbial colonization in the small intestine [33]. Furthermore, bile acids control the growth of cells that line the intestines and impact the makeup of the microbial community in the gastrointestinal tract. Through their antimicrobial properties, particularly when they are predominantly deconjugated, bile acids modulate the functions of various microbial species in the microbiome, weakening bacterial integrity, especially bacterial cell membranes, and impacting the overall population of microbiome [34].

### 2.3. Immunological Barrier

The digestive system is the central location where the body encounters external substances that trigger an immune response, and it is also the largest organ responsible for immune defense. The immune defense system in the intestinal tract primarily comprises gut-associated lymphatic tissue (GALT), which includes organized lymphoid tissues and scattered lymphocytes throughout the intestinal wall [35]. Components of the intestine include Peyer’s patches, lymphoid follicles associated with the mucosa, macrophages distributed in the intestinal mucosa, T helper cells, B cells, plasma cells, and intraepithelial lymphocytes, among other elements [35]. Secretory IgA (sIgA) is the primary component of the intestinal immune system [36]. Lymphocytes and plasma cells, found throughout the intestinal mucosa, are the primary sources of sIgA. Acting as the predominant immunoglobulin in intestinal secretions, sIgA serves as a primary defense mechanism against the invasion of pathogens. Its main functions in the intestinal tract are preventing pathogens from adhering to the intestinal mucosal surface, neutralize toxins produced by bacteria, neutralize viruses, enhancing the phagocytic function of cells with FC receptors, and coordinate with complements and lysozymes for antibacterial activities. A decrease in sIgA levels in the gastrointestinal system can weaken the immune barrier, making it more likely for intestinal bacteria to interact with mucosal epithelial cells, facilitating bacterial translocation and the absorption of endotoxins. Studies have indicated that excess zinc can cause a decline in the quantity of sIgA plasma cells in rats*’* intestinal mucosal lamina propria, impairing their capacity to produce and release sIgA, which ultimately leads to lower levels of mucosal antibody sIgA and a weakened mucosal immune system [37]. This can be considered as one of the underlying factors for intestinal infections or enteric infections. Additionally, besides IgA, intestinal mucosal epithelial cells can release IgE and IgG, along with various other immunoglobulins, that have crucial functions in maintaining intestinal humoral immunity. IgE is essential in the interaction between antigens and intestinal mucosal absorption and is a critical factor in intestinal allergic reactions. Furthermore, IgM plays a protective function in the intestinal mucosa and contributes to the development of inflammatory responses and tissue damage in specific gastrointestinal disorders [38].

### 2.4. Microbial Barrier

Due to its unique structure and physiological role, the digestive system is the primary organ that houses external microbial symbiosis. The symbiotic microbiota in the intestines plays a crucial role in forming the protective barrier of the intestinal mucosa. Bacteria and their community structure can affect the properties of the intestinal mucus barrier by altering intestinal mucosal and systemic metabolic responses that impact human health and disease processes. Intestinal bacteria play a vital role in creating biological barriers, hindering the entry of harmful bacteria, and promoting the growth and maturation of the immune system by producing various enzymes. This process also helps regulate the host’s metabolism [39]. Bifidobacterium bifidum, for instance, contributes to breaking down proteins, fats, and carbohydrates, and produces short-chain fatty acids, which enhance the absorption of vitamins and minerals (such as iron and calcium), thereby boosting sugar and other substances’ metabolism. Additionally, Bifidobacterium and Lactobacillus can maintain normal osmotic pressure in the intestine by promoting mucin expression in caco-2 cells and inhibiting IL-1β-induced NF-κB expression [40]. Furthermore, the gut’s anaerobic microorganisms can break down plant fibers, producing butyric acid, which act as an energy source for intestinal wall cells, facilitating water absorption and maintaining proper metabolism [41]. The application of antibiotics can disrupt the anaerobic flora, leading to an increase in intestinal oxygen content, which can promote the propagation of Salmonella and result in a disease [42]. By addressing the underlying causes and employing microecological agents, such as bifidobacteria, lactobacilli, etc., it is possible to significantly improve the condition and facilitate the restoration of regular intestinal microecological groups [43].

## 3. Assessment of Intestinal Barrier

The various aspects of intestinal barrier function are closely interconnected and inseparable, and they are closely related to the development of NAFLD. A systematic review and meta-analysis statistically investigated the role of intestinal barrier permeability in the pathogenesis of NAFLD. The literature included in this study regarding the detection of intestinal barrier permeability involved a number of different methods, such as oral glucose, 51Cr-EDTA, and zonulin levels [44]. The study concluded that small intestinal permeability was increased in patients with NAFLD and increased with the degree of hepatic steatosis. However, there was no significant difference between patients with simple steatosis and NASH, and it was not associated with hepatic inflammation or fibrosis [44]. Zhuang et al. propose that intestinal permeability correlates with disease severity in NAFLD patients, which may be valuable in predicting the efficacy of metabolic therapies in patients with NAFLD [45]. Therefore, it is necessary to familiarize oneself with the current methods for assessing intestinal barrier function and their main advantages and disadvantages (Table 1).

### 3.1. Evaluation of the Gut Barrier in a Living Organism

#### 3.1.1. Exogenous Inert Probes

To investigate the functional integrity of the intestinal barrier using exogenous inert probes, a permeability test was conducted in a living organism by administering a solute orally, which was later excreted in urine [46]. The method involves the oral administration of inert probes with varying molecular sizes. Currently, commonly used probes include polyethylene glycols (PEGs) [47], monosaccharides such as mannitol and rhamnose [48,49], ^51^Chromium-ethylenediaminetetraacetic acid (^51^Cr-EDTA) [50,51] and FITC-dextran 4000 [52]. Sucrose, a disaccharide, is often used as an indicator of gastroduodenal permeability [53,54]. The technology in this field has advanced significantly, with the current investigation utilizing a multi-sugar identification technique with five separate sugar sensors: sucrose, lactose, l-rhamnose, erythritol, and sucralose, providing precise, site-specific information on gastroduodenal, small, and large intestinal permeability compared to the disaccharides test [55]. However, despite continuous development, this technology still has limitations and drawbacks. The advantage of this method is that it allows the assessment of intestinal permeability in a living organism and provides an overview of intestinal permeability. However, the drawback is that it may not always accurately pinpoint the location of the intestinal barrier dysfunction. Additionally, various factors can influence the assimilation, processing, and elimination of probes, requiring specialized equipment and technology [54,56]. These factors include gastrointestinal motility, intestinal transit time, mucosal blood flow, distribution of biomarkers in the body, drug interference, and renal function. The excretion of ingested substances in urine can also be affected by factors such as urine volume and collection time. Therefore, meticulous monitoring of the experiment and precise measurement of parameters are essential.

#### 3.1.2. Exogenous Inert Probes

In the past ten years, reliable biomarkers have been developed to assess intestinal permeability in the bloodstream, complementing traditional techniques. Zonulin (47 kDa), produced by intestinal and hepatic cells, is currently the only known physiological modulator of intercellular TJ. Disruption of zonulin homeostasis leads to a loosened structure of the intestinal epithelial cell barrier, allowing larger protein molecules to enter the bloodstream [57,58]. Elevated serum zonulin levels have been linked to increased gut permeability, as evidenced by clinical studies showing higher zonulin levels in NAFLD patients compared to healthy controls. This increase in zonulin levels is also associated with the severity of hepatic steatosis [44]. Factors known to disrupt zonulin homeostasis include alcohol-soluble proteins and gliadin proteins in wheat gluten. Additionally, harmful microbes in the gastrointestinal tract can stimulate zonulin production, further compromising the integrity of the intestinal epithelial cell barrier and enhancing intestinal permeability [57,59]. Currently, the detection of zonulin protein primarily relies on commercial ELISA kits.

Calprotectin, a 36 kDa protein complex that binds calcium and zinc, is an additional biomarker for inflammation and intestinal permeability. It consists of one light chain and two heavy peptide chains [60]. Several studies have demonstrated that fecal calreticulin is a highly sensitive marker of inflammation in the intestines [61,62]. The assessment of fecal calreticulin could be utilized as an initial diagnostic tool for individuals presenting symptoms of irritable bowel syndrome or gastrointestinal issues associated with irritable bowel syndrome. It also holds promise for screening inflammatory bowel disease and evaluating patients with irritable bowel syndrome prior to endoscopic procedures and NAFLD/NASH-related impairment of intestinal barrier function [63,64,65,66,67]. However, it has been observed that calprotectin levels are not correlated with the inflammatory condition of the liver and the fibrotic stage of NASH [67]. Therefore, to some extent, calpain is also considered a potential marker for intestinal barrier dysfunction.

FABPs, also known as fatty acid binding proteins, are small proteins with a molecular weight of 15 kDa found in the cytosol of mammalian tissue cells. Currently, at least nine different types of FABPs have been discovered [54]. In the inteseine, FABPs are classified into two types: intestinal type (I-FABP) and liver type (L-FABP) [68]. I-FABP is exclusively present in the gastrointestinal tract, mainly in the cytosol of mature intestinal epithelial cells in the small intestine, accounting for 1% to 2% of cytosolic proteins. Its primary functions include the absorption and transport of fat molecules by the organism, as well as the intracellular redistribution and utilization of fat molecules [68,69]. Due to the short half-life of I-FABP, it is rapidly cleared by the kidneys once it enters the bloodstream, leading to serum levels of I-FABP that are below the detectable limit under normal conditions [70]. In cases of intestinal ischemia or other injuries that increase intestinal permeability, higher levels of I-FABP in the blood are observed and subsequently eliminated by the kidneys. Monitoring the levels of I-FABP in patients’ urine can serve as an early indicator of damage to intestinal barrier function [71]. However, I-FABP alone exhibits high sensitivity in diagnosing specific conditions, such as neonatal strangulated bowel obstruction or necrotizing small bowel colitis [72,73]. For instance, Lau et al. found that plasma I-FABP levels decrease in high-fat fed rats, while I-FABP expression in the jejunum appears to increase, indicating that I-FABP may not be an ideal marker for assessing intestinal barrier function in non-alcoholic fatty liver disease (NAFLD) [74]. Therefore, I-FABP may be more suitable as a marker for assessing obvious damage to the intestinal barrier, such as gastrointestinal cancer, intestinal obstruction, necrotizing colitis, etc., and may not be applicable in the early stages of certain diseases, such as NAFLD-associated intestinal barrier functions.

Citrulline, a non-essential amino acid, is produced by intestinal epithelial cells, released through the basement membrane, and enters the portal vein. It is minimally absorbed by liver cells and primarily reabsorbed and converted into arginine by proximal renal tubular epithelial cells upon entering the systemic circulation. The concentration of plasma citrulline is determined by the efficiency of synthesis in intestinal epithelial cells and conversion by proximal renal tubular epithelial cells. In individuals with normal kidney function, the circulating citrulline level reflects the functional status of intestinal epithelial cells. Research conducted by Crenn et al. revealed that plasma citrulline concentrations in healthy adults typically range between 20 and 60 mmol/L. Concentrations below 20 mmol/L indicate a significant decrease in the number of intestinal epithelial cells [75], suggesting a close relationship between plasma citrulline concentration and the severity of impaired intestinal barrier function [76]. Supplementing with citrulline has been shown to protect against the development of NAFLD by reducing endotoxin translocation to the portal vein. This protective effect is thought to be mediated through the inhibition of the inflammatory response via the TLR4 signaling pathway and increased expression of the tight junction protein claudin-1, which enhances intestinal barrier function [77,76]. However, because citrulline is a non-protein amino acid, its levels may be influenced by the consumption of foods rich in citrulline, such as watermelon [78]. Additionally, certain autoimmune diseases such as rheumatoid arthritis, multiple sclerosis, psoriasis, and systemic lupus erythematosus can interfere with the detection of citrulline [79]. Therefore, measuring circulating citrulline levels can be indicative of gastrointestinal dysfunction when potential interfering factors are taken into consideration.

TFF3, also known as trefoil factor 3, belongs to the trefoil peptide family and is consistently expressed in epithelial tissues, such as the gastrointestinal tract, forming the first line of defense of the intestinal barrier [80]. This process relies on the proper functioning of the tight junction (TJ) barrier and the presence of proteins like claudin-1, zona occludens-1 (ZO-1), and occludin, which are associated with the TJ [81]. Furthermore, as additional studies progress, TFF3 demonstrates a clear protective effect on the mucosa. Despite the unclear understanding of its underlying mechanisms, it increasingly exhibits oncogenic properties and potential effects on the endocrine systems [82]. Recent findings indicate that in pathological conditions such as cancer, colitis, gastric ulcers, diabetes mellitus, and non-alcoholic fatty liver disease [82,83], there are notable alterations in the expression profile and biological impacts of TFF3. Indeed, TFF3 can activate various signaling pathways, such as TLR4 [83], MAPK [84], NF-κB [85], PI3K-AKT [86], STAT3 [87], mTOR [88], and HIF-1α [89], to repair damaged mucosa, regulate glucose and lipid metabolism, which are associated with T2DM, NAFLD as well as other disorders.

### 3.2. Evaluation of the Intestinal Barrier in a Laboratory Setting

Evaluating intestinal barrier function commonly involves using different techniques in live studies. However, these methods are often affected by numerous unidentified factors. On the other hand, in vitro assays can eliminate the influence of these factors and offer more objective and singular research outcomes. Therefore, in vitro assays are particularly beneficial for investigating the fundamental aspects and mechanisms of intestinal physiology. It is not only an important supplement to in vivo assays but also a key research indicator.

#### 3.2.1. Monoculture Models

**Figure 2:**
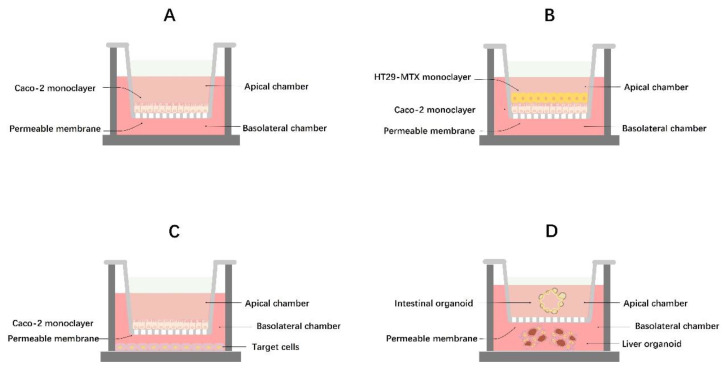
Schematic diagram of the intestinal barrier in a laboratory setting. (**A**) Caco-2 cells were inoculated in the top layer of the chambers. (**B**) Caco-2 and H29-MTX cells were sequentially grown in the apical chamber in appropriate proportions. (**C**) Caco-2 and target cells were grown in the top and bottom chambers, respectively. (**D**) Envisioned intestinal–liver organoid co-culture model.

Caco-2 cell lines are currently the most commonly used for studying intestinal barrier dysfunction. They offer the advantages of a simple structure, broad applicability, and low cost [90]. These cells can mimic the morphology and physiological functions of most cells in the intestine, making them a fundamental choice for research related to gut barrier dysfunction. In Figure 2A, the creation of the Caco-2 cell model is illustrated. The Caco-2 cells are cultured in the apical chamber of a Transwell, and during the culture period, they grow in clusters and differentiate spontaneously. Over time, the top surface of the cells (luminal side) develops a brush border with microvilli. The cells form tight junctions with each other and express various transporter proteins and metabolic enzymes, and ultimately form a layer of polarized enterocytes [91,92]. However, Caco-2 monocytes must be cultured for a specific period and reach a certain density before being used in in vitro gut barrier modeling studies. Generally, as the culture time and cell density increase, the cells exhibit more structural and functional characteristics resembling those of the small intestine. According to Xia et al. [93], once the cells demonstrate differentiation and form complete monolayers on Transwell filter supports, the transmembrane resistance can be monitored using a cellular transmembrane resistivity meter (TEER > 300). This measurement helps determine whether the Caco-2 monoculture model meets the necessary criteria for in vitro intestinal barrier modeling. Utilizing this model, Wang et al. found that tauroursodeoxycholic acid (TUDCA) inhibits intestinal inflammation and barrier disruption in mice with NAFLD [94]. Although the Caco-2 monoculture model is currently the most widely used for studying intestinal barrier dysfunction, it does have limitations. This cell model is static and cannot replicate the dynamic process of intestinal cells, lacks a mucus layer, and is unable to efficiently express all intestinal cell transporter proteins on the surface of the brush border microvilli. This limitation makes it challenging to closely mimic the physiological conditions of human intestinal cells [91]. Therefore, it is crucial to develop a more realistic in vitro cell model that closely resembles actual intestinal cells. This can be achieved by integrating the Caco-2 monoculture model with advanced technological tools and theoretical expertise to meet diverse research requirements.

#### 3.2.2. Co-Culture Models and Intestinal Organoids

Co-culture cell models have been developed to address the limitations of mono-culture cell models. The main models currently being widely used include the Caco-2/HT29-MTX cell co-culture model (Figure 2B) and the Caco-2/target-cell co-culture model (Figure 2C). The Caco-2/HT29-MTX cell co-culture combines the compact cellular arrangement of Caco-2 cells to ensure a functional cell barrier with HT29-MTX cells that secrete mucus to form a protective layer, enhancing the barrier function [95]. Therefore, the Ca-co-2/HT29-MTX cell co-culture model provides a more accurate representation of human intestinal cells’ structural and functional characteristics and physiological conditions. Hoffmann P et al. utilized the Caco-2/HT29-MTX co-culture model to investigate if pathogens release toxins causing infectious gastrointestinal diseases [96]. A recent study demonstrated that the Caco2/HT29-MTX co-culture model showed superior functionality in terms of fatty acid uptake and release compared to the Caco-2 intestinal barrier, which is linked to the pathogenesis of NAFLD [97]. However, adjustments are necessary based on the actual cell ratios and conditions of directional culture of HT29-MTX cells, as the growth rates of the two cell types differ during establishment, producing varying experimental outcomes [98].

The Caco-2/target-cell co-culture model is primarily used to explore the immune response of intestinal cells and its relation to the function of the intestinal barrier. Through the use of a Caco-2/HepG2 cell co-culture model, researchers found that alginate was less likely to induce intracellular fat accumulation compared to glucose, maltose, trehalose and inulin, suggesting that alginate may be more beneficial to individuals with NAFLD [99]. Camille et al. identified that the Caco-2/TC7 cell line, derived from human cells, serves as an effective in vitro model for studying the molecular mechanisms of TICE enterocytes. This model aids in identifying molecular targets in the gut that could potentially facilitate cholesterol transport reversal and combat dyslipidemia associated with NAFLD/NASH development [100]. Hoki T et al. discovered that hepatic iron accumulation in NASH patients is mainly due to the increased expression of divalent metal transporter 1 (DMT1) secreted by the Ca-co-2/TC7 cell lines, activating iron-regulated protein (IRP) [101]. Although co-culture models can partially address the limitations of monoculture models, they come with drawbacks such as strict culture conditions (cell ratio, specific medium components, cell contamination), complex procedures (frequent TEER monitoring, observation and differentiation characteristics), and numerous unpredictable factors. Furthermore, co-culture models are restricted to horizontal interactions and cannot fully replicate the three-dimensional barrier function of the intestine.

Conversely, organoid models can overcome these limitations. Organoid technology has emerged as a powerful tool for investigating diseases and their progression. A key feature of organoid cultures is the presence of both stem and specialized cell lines that spontaneously form complex three-dimensional structures resembling key histological and functional aspects of living tissues. Organoids have been extensively utilized in drug screening, disease modeling, and studying host–microbe interactions. However, the use of intestinal organoids to study NAFLD/NASH has not been reported. Unlike liver organoids, it remains uncertain whether intestinal organoids derived from obese/fatty liver patients can maintain the NAFLD/NASH phenotype [102]. To address the challenges of using organoid technology to study the intestinal barrier in NAFLD/NASH, there is optimism for the development of an intestinal–liver organoid co-culture model (Figure 2D) in the near future, which may offer significant advancements [102]. In a similar vein, through the application of microfluidic chip technology, researchers have designed a microfluidic multi-class organ system to investigate human liver–islet axis insulin and glucose regulation in normal and diseased conditions [103].

## 4. Gut Dysbiosis and Gut–Liver Axis

Under normal circumstances, various microbial communities coexist in the gut in specific proportions, maintaining gut microbiota homeostasis and participating in physiological processes such as metabolism and immune function. Clinical research indicates that patients with NAFLD exhibit an imbalance in the composition and types of gut microbiota compared to healthy individuals [104]. Transplanting gut microbiota from NAFLD patients to germ-free mice can induce fatty livers, while transplanting gut microbiota from obese children who have undergone dietary weight loss to germ-free mice does not cause significant changes [105,106]. Human studies have shown an increase in the relative abundance of Proteobacteriaceae (Proteobacteria), Enterobacteriaceae, Escherichia, and Doreia in NAFLD patients, while Rumenococcaceae, Faecalibacterium Praeplastrum (Ruminococcaceae), Faecalibacterium Prausnitzii, Coprococcus, Eubacterium, Prevotella, and Anaerospacteracter were found to decrease in relative abundance [107,108,109,110]. In rodent trials, individuals with NAFLD showed a significant increase in Firmicutes abundance, while Bacteroidetes were reduced [111]. Dysbiosis can lead to NAFLD through various pathways [112].

Initially, alterations in gut microbiota impact host metabolism through the production of short-chain fatty acids (SCFAs). SCFAs are the result of colonic bacterial fermentation of polysaccharides and include substances such as acetate, propionate, and butyrate. Experimental models of NAFLD and studies involving obese individuals have shown elevated levels of SCFAs [113,114]. These fatty acids can activate specific G-protein-coupled receptors, namely GPR41 and GPR43, found in all organs involved in NAFLD development, including adipose tissue, liver, and intestines. Activation of these receptors by SCFAs can trigger de novo lipogenesis, cholesterol synthesis, and disruptions in glucose regulation [115]. Additionally, SCFAs influence food consumption by affecting neuronal control of hunger [116].

Another critical way in which the gut microbiota contributes to NAFLD is by directly altering lipid metabolism. Bacteria have been found to suppress the production of fasting-induced adipocyte factor, a key inhibitor of lipoprotein lipase (LPL). This suppression leads to an increased release of free fatty acids from very low-density lipoprotein particles into the liver, promoting steatosis. Research provides compelling evidence for the causal and potential mechanisms of gut microbiota in the development of fatty liver, linking disturbances in gut microbiota to NAFLD development [117].

A third critical pathway through which dysbiosis contributes to NAFLD development involves compromising the intestinal barrier. This compromise facilitates the movement of bacteria or their by-products, such as lipopolysaccharides (LPSs), into the bloodstream, a process associated with the progression from NAFLD to non-alcoholic steatohepatitis (NASH) in both experimental and human studies [117,118]. The breach in the intestinal wall’s defensive layers, resulting from a breakdown in the protective mechanisms operating at various levels of the intestinal barrier, allowing for the translocation of these microbial elements, plays a pivotal role in advancing from NAFLD to NASH. Tight junctions (TJs) form a selectively permeable barrier between neighboring epithelial cells [119,120], but diet-induced obesity can disrupt their expression and arrangement, leading to increased intestinal permeability [121]. Dysbiosis has been observed to diminish the expression of antimicrobial peptides (AMPs) and cytokines by specific immune cells, a condition that correlates with the onset of NASH [122]. Restoring the disrupted intestinal barrier function and dysbiosis is a potential strategy for managing NAFLD.

Notably, clinical observations and basic research have shown that intestinal barrier damage rarely leads to liver injury alone, but can exacerbate pre-existing liver diseases, such as NAFLD and drug-induced liver injury. This evidence suggests the existence of a hepatic barrier in the gut–liver axis that protects the liver from enterogenic pathogenic factors. Moreover, people found that liver sinusoidal endothelial cells (LSECs) take up and clear viruses, phages, microbial products, and metabolic waste. LSECs also maintain the homeostasis of the hepatic immune environment through tolerance-inducing and anti-inflammatory functions under physiological conditions. In contrast, under pathological conditions, the clearance function of LSEC is impaired and triggers the pro-inflammatory mode. Therefore, LSECs have been proposed as a hepatic barrier to the gut–liver axis, which protects the liver against gut-derived pathogenic factors [123].

## 5. Inflammation and TLR Signaling

It is now evident from numerous studies that a high blood endotoxin level is closely associated with the development of NAFLD. According to a recent systematic review and meta-analysis, the findings endorse using blood endotoxin levels as a significant diagnostic biomarker for NAFLD and are applicable to identifying the disease and determining the stage of disease progression. In addition, it also suggests that blood endotoxin levels could indicate increased intestinal permeability in NAFLD [124]. Lipopolysaccharide (LPS), a key component of endotoxin, plays a crucial role in this mechanism as it is found in the outer cell wall of Gram-negative bacteria. Research indicates that individuals who are overweight or have NAFLD exhibit abnormal proliferation in the small intestine (known as enteric bacterial overgrowth syndrome or EBOS) compared to individuals with a healthy weight [125]. This leads to a rise in the number of bacteria that contain lipopolysaccharide (LPS), which can activate adenosine cyclase in the intestinal mucosa, causing damage to epithelial cell mitochondria and lysosomes, resulting in villous cell necrosis, epithelial cell autolysis, local intestinal mucosal damage, and increased intestinal permeability [122]. Endotoxin travels through the bloodstream and reaches the liver via enterohepatic circulation. In the liver, Toll-like receptors (TLRs) in Kupffer cells or hepatocytes recognize and bind to the endotoxin, triggering the release of various cytokines. This process activates immune-inflammatory responses and contributes to liver inflammation and pathological damage.

TLRs are a group of pattern-specific and widely distributed receptors that play a crucial role in the activation of the innate immune system by recognizing pathogen-associated molecular patterns (PAMPs) as well as endogenous molecules derived from dying host cells, known as damage-associated molecular patterns (DAMPs) [126]. When TLRs bind to their corresponding ligands, they activate signaling pathways through the common signal adaptor molecule Myeloid differentiation primary response 88 (MyD88), which is shared by interleukin-1 (IL-1) receptors and all TLRs except TLR3. These events ultimately lead to the activation of NF-κB, p38, and c-Jun N-terminal kinase (JNK), as well as the production of inflammatory cytokines such as tumor necrosis factor-alpha (TNF-α) and interleukin-6 (IL-6) [127]. Moreover, TLR recognition by microbial motifs enhances intestinal epithelial barrier function by inducing the tightening of intercellular junctions, secretion of mucus and antimicrobial peptides (AMPs), and production of reactive oxygen species (ROS) [128]. However, it has also been shown that symbiotic enterobacteria weaken the intestinal barrier by activating the intestinal epithelial innate immune receptor TLR-2 signaling and downregulating neuropilin-1 (NRP1) and Hedgehog signaling regulated by it in the intestinal epithelium [129], indicating its complex roles in intestinal barriers.

**Figure 3:**
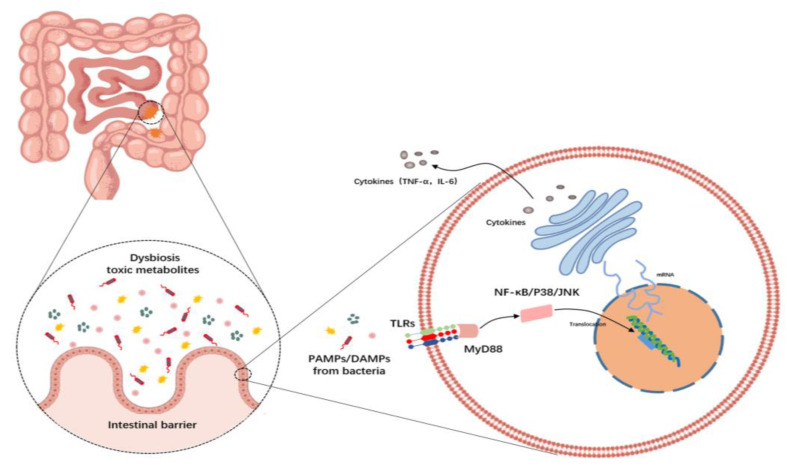
Possible mechanisms for the role of TLR signaling and related inflammatory signaling pathways in the development and progression of NAFLD due to gut-liver barrier dysfunction. Pathogen-associated molecular patterns (PAMPs) or damage-associated molecular patterns (DAMPs) are recognized by TLRs, thereby activating NF-κB, p38, and c-Jun N-terminal kinase (JNK) signaling pathways, which initiates the production of inflammatory cytokines, such as tumor necrosis factor-alpha (TNF-α) and interleukin-6 (IL-6).

To date, more than ten members of the TLR family (TLR1-10) have been identified in mammals. These members are distinguished by their type I transmembrane proteins, featuring an extracellular domain rich in leucine and a cytoplasmic tail accountable for ligand recognition [126,130]. Among them, the significance of TLR4 and gut-derived LPS in the animal model of NASH has been emphasized in numerous studies [131,132]. Additionally, translocated bacterial DNA binds to TLR9 on Kupffer cells in choline-deficient amino acid-defined (CDAA) diet-induced NASH mice, leading to the synthesis of IL-1β, which prompts lipid accumulation and cell death in hepatocytes while triggering the activation of hepatic stellate cells (HSCs) to cause liver fibrosis [133]. These findings indicate that the activation of TLR9 signaling plays a vital part in developing and advancing NAFLD to NASH. In addition to TLR4 and TLR9, by employing a CDAA diet-induced NASH mice model, TLR2 also has a crucial function in the progression of NASH. However, simple hepatic steatosis induction occurs independently of TLR2 signaling [134,135], indicating its importance in the advancement of NAFLD to NASH rather than in the early stages of NAFLD development. These conclusions imply that the activation of TLR2, TLR4, TLR9 and MyD88 along with downstream inflammatory signaling pathways are crucial in the development and progression of NAFLD to NASH (Figure 3).

## 6. Therapeutic Strategies Targeting Gut Permeability and Gut Microbiota

NAFLD patients face an increased risk of overall morbidity and mortality, driven primarily by liver complications, cardiovascular diseases, and cancer. The treatment and management of NAFLD primarily target liver diseases, particularly fibrosis, which is an important prognostic factor, Additionally, addressing metabolic comorbidities such as obesity, type 2 diabetes, and dyslipidemia is essential. Medical conferences universally advocate for weight loss through lifestyle modifications, including a balanced diet and regular physical activity as the cornerstone of NAFLD treatment. Despite the challenging nature of implementing and sustaining lifestyle changes, pharmacological interventions should be considered for individuals with progressive disease, specifically those with non-alcoholic steatohepatitis (NASH) and fibrosis, while taking into account safety concerns and NAFLD comorbidities. Despite the ongoing exploration of various treatment modalities (e.g., anti-obesity, antidiabetic, and lipid-lowering medication), no drugs have yet been approved for NAFLD specifically.

### 6.1. Probiotics

According to the FAO and WHO, probiotics are living microorganisms that provide health advantages to the host when given in adequate amounts [136], including conventional probiotics (CPs) and next-generation probiotics (NGPs) (Table 2).

#### 6.1.1. Conventional Probiotics

The predominant types of probiotics, commonly referred to as conventional probiotics (CPs), include Lactobacillus, Bifidobacterium, Clostridium casei, Lactobacillus acidophilus, Actinobacillus and Saccharomyces cerevisiae [137]. Jang et al. discovered that the probiotic Lactobacillus rhamnosus GG can compete with the host for intestinal fatty acids, resulting in the prevention of hepatic steatosis caused by a high-fat diet (HFD) [138]. The probiotic blend reduces weight, triglycerides, total cholesterol, and LDL-C levels, while increasing HDL-C levels, leading to a decrease in hepatic steatosis and inflammatory cell infiltration in rats used in experiments. This ultimately reduces hepatic lipid accumulation, relieves hepatic inflammation, and improves NAFLD [139]. A formulation based on Lactobacillus johnsonii (*L. johnsonii*) BS15 has been shown to protect high-fat diet-fed NAFLD mice from hepatic steatosis and hepatocyte apoptosis by reducing intestinal permeability, altering gut microbiota composition, lowering LPS levels, and downregulating liver inflammatory factors such as TNF-α [140]. Another study demonstrated that oral administration of Lactobacillus paracasei N1115 alleviates HFD-induced hepatic steatosis and the release of the inflammatory factor tumor necrosis factor (TNF-α), thereby slowing down the progression of liver fibrosis [141]. In separate research, treatment with Lactobacillus paracasei inhibited the pro-inflammatory M1 Kupffer cell response and promoted the anti-inflammatory M2 response, resulting in a significant reduction in the expression levels of pro-inflammatory cytokines TNF-α and Monocyte chemoattractant protein-1 (MCP-1) [142]. Additionally, intervention with Saccharomyces boulardii, a strain of probiotic yeast, improved hepatic steatosis in HFD-induced NAFLD rats and decreased the expression of TNF-α [143]. According to the research, probiotics preserve the integrity of the intestinal epithelial barrier, reduce the translocation of pathogens across the intestinal epithelial barrier, and relieve the muscle tension disorder response caused by HFD. Consequently, they can mitigate the effects of HFD, slow down the progression of NAFLD, and ultimately alleviate its impact [144]. These findings are significant in advancing the utilization of probiotics and gut microbiota as potential targets for NAFLD treatment.

#### 6.1.2. Next-Generation Probiotics

The discovery of a new group of probiotics, called next-generation probiotics (NGPs) such as Akkermansia muciniphila, Faecalibacterium Prausnitzii, Bacteroides uniformis, Bacteroides xylanisolvens, Bacteroides fragilis, Eubacterium hallii and Propionibacterium spp., has been enabled by advancements in cultivation methods, sequencing technologies, and bioinformatics methods. Certain members of Clostridium spp. IV, XIVa, and XVIII have been shown to improve the management of NAFLD [145]. According to the current studies [145,146], NGPs differ primarily from conventional probiotics in the following several ways:

1. Strain selection: NGPs mostly derived from commensals and that belong to diverse genera, which are selected based on advanced screening techniques, including genomics, metagenomics, and functional assays, that allow for identifying and isolating strains with specific beneficial properties, such as enhanced colonization, improved survival in the gastrointestinal tract, and targeted health benefits.
2. Precision and personalized approach: NGPs are designed to be more precise and personalized in their application. They can be tailored to address specific health conditions or individual needs, considering factors like microbiome composition, genetic background, and lifestyle factors.
3. Mechanistic understanding: NGPs are characterized by a better understanding of their mechanisms of action. Advances in molecular biology and omics technologies have enabled researchers to uncover the molecular interactions between NGPs and the host, providing insights into the underlying mechanisms through which they exert their beneficial effects.
4. Therapeutic potential: NGPs have the potential to be used as therapeutic agents for various diseases beyond gut health. By manipulating the gut microbiota and engaging with the host’s immune system, they can address ailments like metabolic disorders, inflammatory conditions, and neurological disorders.
5. Combination therapies: NGPs can be combined with other therapies, such as pharmaceutical drugs or dietary interventions, to enhance their efficacy. This approach, known as synbiotics, involves the synergistic interaction between NGPs and other therapeutic agents to achieve improved health outcomes.

Therefore, NGPs have the potential to be a valuable tool for personalized medicine, as they can target specific diseases by modulating the gut microbiota. Akkermansia muciniphila, a bacterium that degrades mucin, is a prevalent species in the human gut microbiota, constituting 0.5% to 5.0% of the overall bacterial population [147]. NAFLD mice exhibit a notable decrease in the abundance of Akkermansia muciniphila compared to wild-type mice, and interventions that enhance its abundance have shown improvements in metabolic parameters [148]. Fructo-oligosaccharides, similar to prebiotics, can elevate the levels of Akkermansia muciniphila and alleviate the dysregulation of associated diseases [149,150]. Despite not causing significant changes in the gut microbiota of diet-induced obese mice, the use of Akkermansia muciniphila can counteract metabolic disorders induced by a high-fat diet (HFD), including elevated adipose tissue mass, metabolic endotoxemia, inflammation in adipose tissue, and insulin resistance, all of which are closely linked to the development of NALFD/NASH. This indicates that Akkermansia muciniphila has the potential to prevent or treat obesity and associated metabolic disorders [150]. Further research has revealed that the primary by-products of Akkermansia muciniphila, including propionic acid, and acetic acid, can affect the expression of genes involved in host lipid metabolism, as well as their epigenetic regulation [151]. Similarly, Bacteroides have a similar effect by degrading indigestible dietary fibers, producing a significant quantity of short-chain fatty acids, primarily acetate and propionate [152]. Gauffin Cano et al. have demonstrated that in HFD-fed mice, a single strain of Mycobacterium anisopliae CECT 7771 can reduce body weight and alleviate steatosis by reducing cholesterol levels in the liver and serum [153]

### 6.2. Fecal Microbiota Transplantation

In addition to supplementing probiotics, fecal microbiota transplantation (FMT) has emerged as a novel therapeutic approach in recent years. The procedure involves transferring the operational intestinal bacteria from a fecal sample of a healthy person into the patient’s intestines to rebuild a healthy gut microbiota, thereby treating both intestinal and extraintestinal conditions. FMT has been demonstrated to effectively manage refractory and recurrent Clostridium difcile infection (CDI) [154] and shows potential for addressing gastrointestinal and extraintestinal disorders [155,156]. It has also been shown to alleviate liver inflammation in a high-fat diet-induced mouse model of NASH by reducing lipid accumulation in the liver and lowering pro-inflammatory cytokine levels in the bloodstream [156]. Studies [157] suggest that FMT can help reduce fat accumulation in the liver and alleviate fatty liver disease by restoring balance to gut microbiota. Furthermore, FMT has proven to be more effective in gut microbiota reconstruction for lean NAFLD patients compared to obese NAFLD patients [158]. However, some issues still need to be addressed in the clinical application of FMT. Firstly, current transplant techniques can be conducted through various routes, including nasogastric or nasoenteric tubes, upper gastrointestinal endoscopy (esophagogastroduodenoscopy), colonoscopy, or retention enema. Nevertheless, systematic reviews indicate that these methods have varying efficacy and are often associated with variability in the donor FMT material [159]. Therefore, further standardization of the fecal microbiota transplantation process is necessary. Secondly, there is significant heterogeneity in the provided FMT material. Factors contributing to this heterogeneity include the weight of the donor feces, the volume of the preparation solution, and the method of preparing the FMT material. It has been observed that FMT prepared with different solvents (e.g., water, saline, milk, or other diluents) can have varying therapeutic effects [160]. Given that FMT is a unique biological drug derived from the human body but not classified as organ transplant, strict monitoring and preservation of donor specimens are required for the widespread application of FMT. Moreover, there is a need for further improvement in the relevant regulatory agencies and laws [161,162,163].

### 6.3. Lifestyle Changes

#### 6.3.1. Exercise

In modern society, a growing number of individuals have embraced a sedentary lifestyle, leading to a rise in the prevalence of metabolic disorders such as type 2 diabetes, obesity, cardiovascular illness, and NAFLD. A recent study has found that regular physical activity boosts the digestive system, resulting in positive reactions and enhancing the strength of the intestinal barrier [164]. Moderate physical activity in mice has been shown to alleviate damage to the intestinal barrier caused by chronic stress, reduce bacterial translocation, and maintain intestinal permeability [165]. Research has indicated that women who engage in regular physical activity possess a wider range of beneficial microbial communities, including Faecalibacterium prausnitzii, Roseburia hominis, and Akkermansia muciniphila, compared to inactive women [166]. Additional research has uncovered that these microorganisms are recognized as butyrate producers and positively impact maintaining the integrity of the intestinal barrier, regulating the host’s immune system, and controlling lipid metabolism [167,168,169]. Similar results have been observed in animals, with mice undergoing physical exercise exhibiting an increase in symbiotic groups such as Bifidobacterium, Lactobacillus, and Akkermansia muciniphila [170,171]. In average athletes, moderate exercise has been associated with positive effects on health, including decreased inflammation and intestinal permeability, as well as improvements in body composition [172]. During exercise, a significant amount of lactates is released and secreted into the gut, altering the pH levels. Conversely, high-intensity exercise may have adverse effects on gut function. Following vigorous physical activity, approximately 70% of athletes may experience symptoms such as stomach aches, nausea, and bowel irregularities [173,174]. Extended physical activity can lead to a decrease in microbial diversity, an increase in the quantity of Helicobacter pylori, and the onset of heightened permeability in the intestines, allowing bacteria and their harmful substances to enter the bloodstream, triggering widespread inflammation [175,176]. Animal studies have shown that intense acute endurance exercise can induce changes in intestinal permeability [177].

#### 6.3.2. Dietary Carbohydrates

The study of the impact of dietary elements on the maintenance and operation of the intestinal barrier is gaining popularity. Carbohydrates are organic substances composed of carbon, hydrogen, and oxygen. They are highly prevalent, exhibiting various chemical structures and biological roles. Consumption of simple carbohydrates such as sugars like sucrose and fructose has been observed to cause rapid changes in the microbiota, leading to disruptions in the host’s metabolism [178,179]. Cho et al. reported that fructose-induced nitrosylation of intestinal barrier junction proteins results in gut leakage, resulting in hepatic steatosis and fibrosis [180]. In contrast, complex carbohydrates, specifically polysaccharides and dietary fiber that can be broken down by specific microbiota, act as a source of nourishment for the diverse microbiota present in the gut environment. This has significant effects on the ecology and well-being of gut microbes. [181]. Diets rich in polysaccharides are associated with increased diversity in the gut microbial community and support the growth of beneficial bacteria like Akkermansia, Bifidobacteria, and Lactobacillus. Additionally, gut microbiota can utilize intermediate oligosaccharides to produce short-chain fatty acids beneficial to the host. For instance, Dendrobium polysaccharides (DOPs) are not easily digested and absorbed but stimulate the production of butyric acid by gut microorganisms, particularly by Parabacteroides sp. HGS0025, resulting in improved gut health and immune function [182,183]. By acting on Akkermansia muciniphila, DOPs intervention also stimulates mucin production, ultimately enhancing intestinal barrier function [184].

#### 6.3.3. Dietary Proteins

Dietary protein is another crucial macronutrient, and individuals must consume a specific amount of protein daily to obtain amino acids and the necessary nitrogen to synthesize tissue proteins. The relationship between protein consumption and well-being follows a U-shaped pattern. Inadequate protein intake is associated with malnourishment, while excessive intake beyond the acceptable threshold is linked to overnutrition-related ailments [185]. Studies have indicated that following a protein-rich diet may decrease the presence of microorganisms like Lachnospiraceae, Ruminococcaceae, and Akkermansia, which could potentially contribute to the development of specific illnesses [186]. Additionally, gut bacteria can metabolize proteins, especially those found in red and processed meats containing L-carnitine and choline. This metabolism results in the production of trimethylamine (TMA), which is subsequently converted into trimethylamine N-oxide (TMAO) [187]. Elevated TMAO levels are associated with a higher risk of various metabolic disorders like NAFLD/NASH [188].

#### 6.3.4. Dietary Fats

High-fat diets are harmful to health. According to recent reports, there is a potential link between a diet rich in fats and the occurrence of endotoxemia and barrier dysfunction [189]. The constituents in the food interact closely with epithelial cells and serve as the primary triggers for modifying the intestinal barrier [190]. Moreover, a high-fat diet reduces beneficial bacteria and SCFAs, interferes with bile acid metabolism, induces inflammation, and exacerbates further deterioration of NALFD to NASH [191].

## 7. Conclusions

While the detailed structure and role of the epithelial barrier have been extensively explained, the precise collaboration and underlying mechanisms among its various constituents are still not fully understood. The significance of gut barrier function is deemed crucial, yet clinical trials are absent, and there is no supporting evidence concerning NAFLD/NASH. On the other hand, NGPs and conventional probiotics have been found to improve gut barrier function, but no related biopharmaceuticals have been developed and applied in clinical settings. The next step of research should focus on clinical studies of NAFLD, evaluating the gut microbiota function profiles and corresponding protein metabolites, correlating them with NAFLD/NASH pathological characteristics, changes in gut permeability, and related mechanisms. In the end, clarifying the function and mechanisms of the intestinal barrier in different phases of NAFLD can offer fresh perspectives for treating NAFLD/NASH.

## Figures and Tables

**Table 1:** Overview of the different methods/models used for the assessment of the intestinal barrier in NAFLD/NASH.

Methods/Models		Advantages	Disadvantages	Reference
In Vivo				
Exogenous inert probes	Polyethylene glycols (PEG), ^51^Chromium-ethylenediaminetetraacetic acid (^51^Cr-EDTA), FITC-dextran 4000, monosaccharides, disaccharides	(1) Non-invasive via oral administration (2) Multi-sugar gives an overview of the intestinal permeability	(1) Non-specific and easily disturbed by multiple factors. (2) Cannot always accurately assess the location of intestinal barrier dysfunction. (3) Requires special equipment and technology.	[43,47,49,53]
	Multi-sugar			[52]
Endogenous biomarkers	Zonulin	Easily assessed by low equipment and method requirements	Non-specific and easily interfered with by other factors.	[43]
	Calprotectin	(1) Non-invasive via fecal testing (2) Easy to use in clinical trials	Independent of the hepatic inflammatory state and fibrosis stage of NASH.	[64,65,66]
	Fatty acid binding proteins (FABPs)	(1) Specific and sensitive (2) Non-invasive via urine testing (3) Easy to use in clinical trials	Not recommended for assessing intestinal barrier function in NAFLD and a low limit of detection, as well as a short half-life time.	[67,73]
	Citrulline	Higher specificityand sensitivity to small intestinal permeability	Mainly for small intestinal permeability and easily interfered with by other factors.	[75,76]
	Trefoil factor 3 (TFF3)	Mechanisms closely related to intestinal barrier maintenance	Low specificity and easily interfered with by other factors.	[82]
Monoculture	Caco-2 cells	Simple structure, broad applicability, and low cost	Must be cultured for a certain time and to reach a certain density before being used and cannot simulate the dynamic process of the intestinal barrier.	[93]
Co-culture	Caco-2/HT29-MTX cells Caco-2/HepG2 cells Caco-2/TC7 cells	More accurate replication of the structural and functional characteristics of the human intestinal barrier and its physiological environment	Strict culture conditions, complex procedures, many uncertain factors, limited to horizontal interactions and cannot fully replicate the three-dimensional barrier function of the intestine.	[96,98,99,100]
Organoids	Derived from obese/fatty liver patients or laboratory animal	Form complex three-dimensional structures resembling important histological and functional aspects of living tissues	(1) Unstable for maintaining the NAFLD/NASH phenotype. (2) Gut–liver organoid co-culture model remains elusive.	[101]

**Table 2:** Overview of the different probiotics in improving NAFLD.

Organism	Effects	References
Conventional probiotics (CPs)		
Lactobacillus rhamnosus GG	hepatic steatosis ↓	[138]
Probiotic blend (*B. longum*, *B. lactis*, and *B. breve* and *L. reuteri* and *L. plantarum*)	body weight ↓ triglycerides ↓ total cholesterol ↓ LDL-C ↓and HDL-C ↑	[139]
*L. johnsonii* BS15	intestinal permeability ↓ LPS levels ↓ liver inflammatory factors ↓	[140]
Lactobacillus paracasei N1115	hepatic steatosis ↓ the inflammatory factors ↓	[141]
Lactobacillus paracasei	pro-inflammatory cytokines ↓	[142]
Saccharomyces boulardii	hepatic steatosis ↓ TNF-α ↓	[143]
Akkermansia muciniphila	adipose tissue mass ↓ metabolic endotoxaemia ↓ adipose tissue inflammation ↓ insulin resistance ↓	[148]
Bacteroides	SCFAs ↑	[151]
Mycobacterium anisopliae CECT 7771	body weight ↓ steatosis ↓	[152]
